# A new species of deep-water spider crab of the genus *Paramaya* De Haan, 1837 from the Bay of Bengal, India (Crustacea, Brachyura, Majidae)

**DOI:** 10.3897/zookeys.769.26152

**Published:** 2018-06-26

**Authors:** Peter K. L. Ng, M. Prema, S. Ravichandran

**Affiliations:** 1 Lee Kong Chian Natural History Museum, 2 Conservatory Drive, National University of Singapore, Singapore 117377, Republic of Singapore; 2 Centre of Advanced Study in Marine Biology, Annamalai University, Parangipettai 608 502, India

**Keywords:** deep-water, Indian Ocean, Majoidea, new species, taxonomy, spider crab

## Abstract

The identity of the majid species of *Paramaya* De Haan, 1837, in the Indian Ocean is clarified with the collection of fresh specimens from the Bay of Bengal. Previously identified as *P. spinigera* (De Haan, 1837) which is known only from Japan, Taiwan, and Korea, the material from eastern India is here referred to a new species, *P. mulli* **sp**. **n.** The new species can easily be distinguished from all congeners by its relatively shorter pseudorostral and carapace spines, more swollen branchial regions, distinctly granulated male thoracic sternum, and the G1 is not prominently curved with the dorsal projection on the sub distal part short and the tip rounded.

## Introduction

Ng and Richer de Forges (2015) revised the majid genus *Maja* Lamarck, 1801, and recognised *Paramaya* De Haan, 1837, as a valid taxon from the Indo-West Pacific. Three species of *Paramaya* are currently known: *P. coccinea* Ng & Richer de Forges, 2015 [Vanuatu], *P. ouch* Ng & Richer de Forges, 2015 [Philippines], and *P. spinigera* (De Haan, 1837) [Japan, Taiwan, and Korea] (Ng and Richer de Forges 2015; Ko and Lee 2015). There is a record of *Paramaya spinigera* from Beluchistan in Pakistan in the Indian Ocean by Alcock (1895) and Alcock and Anderson (1898) with Ng and Richer de Forges (2015) recording a specimen from Sri Lanka. None of these specimens, however, could be examined, and Ng and Richer de Forges (2015: 156) noted that “although on the basis of geography, they are probably different species [from the Pacific ones]. For the moment, we do not know their precise identities.”

Collections from deep-water ports in India have obtained numerous interesting new brachyurans over the last few years (Ng and Kumar 2015, 2016, Mendoza and Suvarna Devi 2017, Ng et al. 2017a–c, Prema et al. 2018; Ng et al. 2018) and pair of *Paramaya* were recently obtained from Pazhayar, near Chennai in eastern India. These specimens now allow resolving the identity of the Indian Ocean *Paramaya*. Not surprisingly, they represent a new species, and though superficially similar to *Paramaya spinigera* s. str., it nevertheless differs from congeners in various carapace features, ambulatory leg proportions as well as the structures of the male thoracic sternum and gonopods. They are here described as *Paramaya mulli* sp. n.

## Material and methods

The terminology used in this paper follows Ng and Richer de Forges (2015) and Davie et al. (2015), and the measurements provided (in millimetres) are of the post-pseudorostral carapace length (from the base of spines to the posterior carapace margin, not including median posterior spines) against the maximum carapace width, respectively. Specimens examined are deposited in the Centre of Advanced Study in Marine Biology, Annamalai University (CASAU), Parangipettai, Tamil Nadu, India; and the Zoological Reference Collection of the Lee Kong Chian Natural History Museum (ZRC), National University of Singapore.

## Systematics

### Family Majidae Samouelle, 1819

#### Genus Paramaya

Taxon classificationAnimaliaDecapodaMajidae

De Haan, 1837

##### Type species.

Pisa (Paramaya) spinigera De Haan, 1837; by monotypy.

#### Paramaya mulli sp. n.

Taxon classificationAnimaliaDecapodaMajidae

http://zoobank.org/694A5779-FC41-4105-B536-9FB7DC23D65D

**Figure 1.**
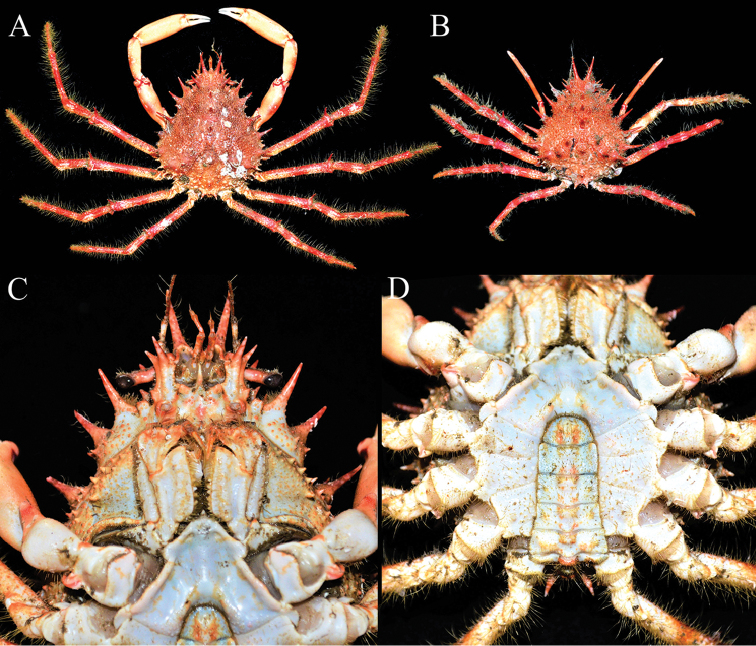
Colours in life. *Paramaya mulli* sp. n. **A, C, D** holotype male (70.4 × 61.4 mm) (CASAU), India **B** paratype female (40.0 × 33.5 mm) (CASAU), India **A, B** overall habitus **C** buccal cavity, epistome, antennae and antennules **D** thoracic sternum and pleon.

**Figure 2.**
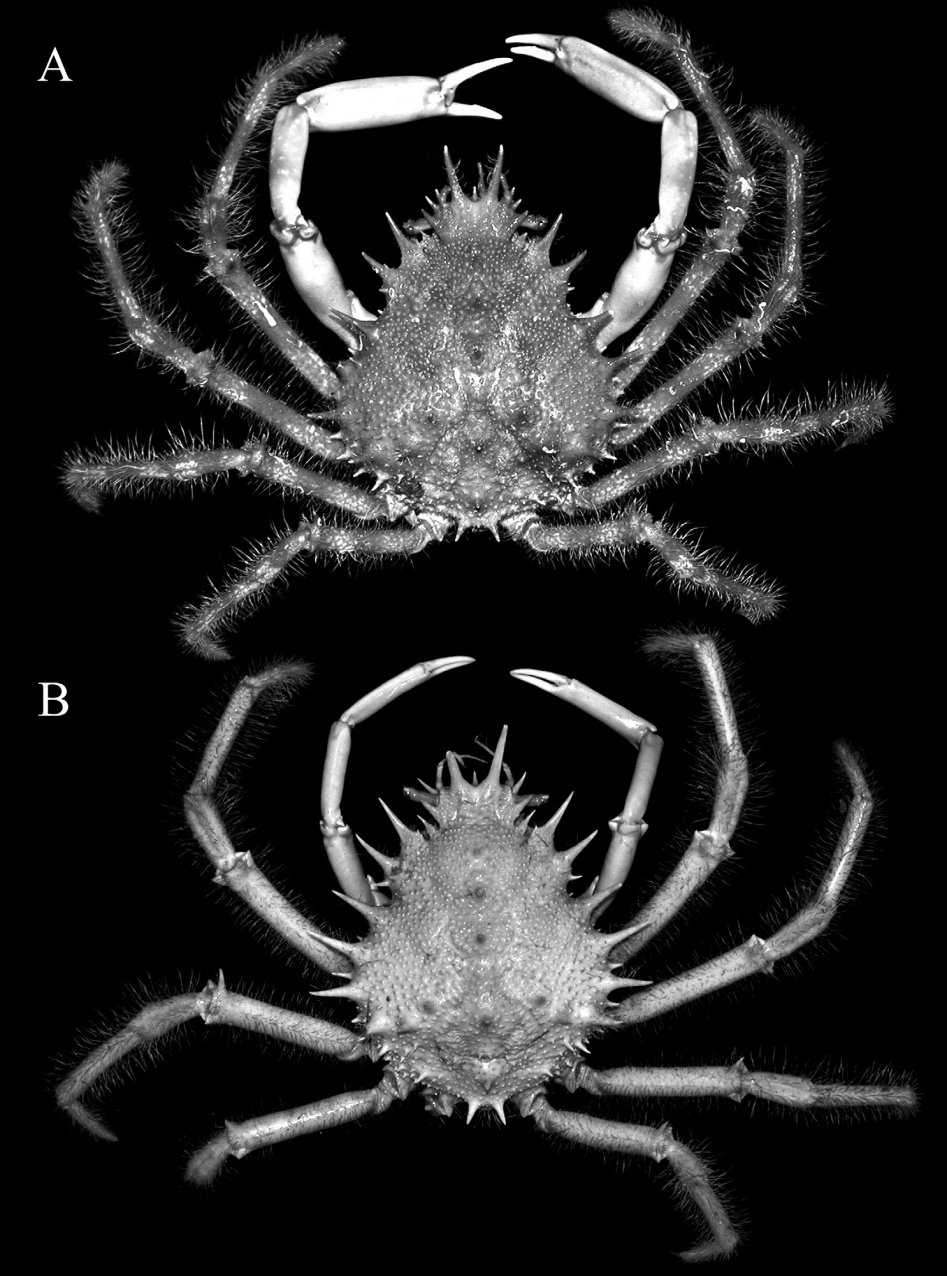
Overall habitus **A** *Paramaya mulli* sp. n., holotype male (70.4 × 61.4 mm) (CASAU), India **B** *P. spinigera* (De Haan, 1837), male (73.6 × 55.3 mm) (ZRC 1999.738), Taiwan.

**Figure 3.**
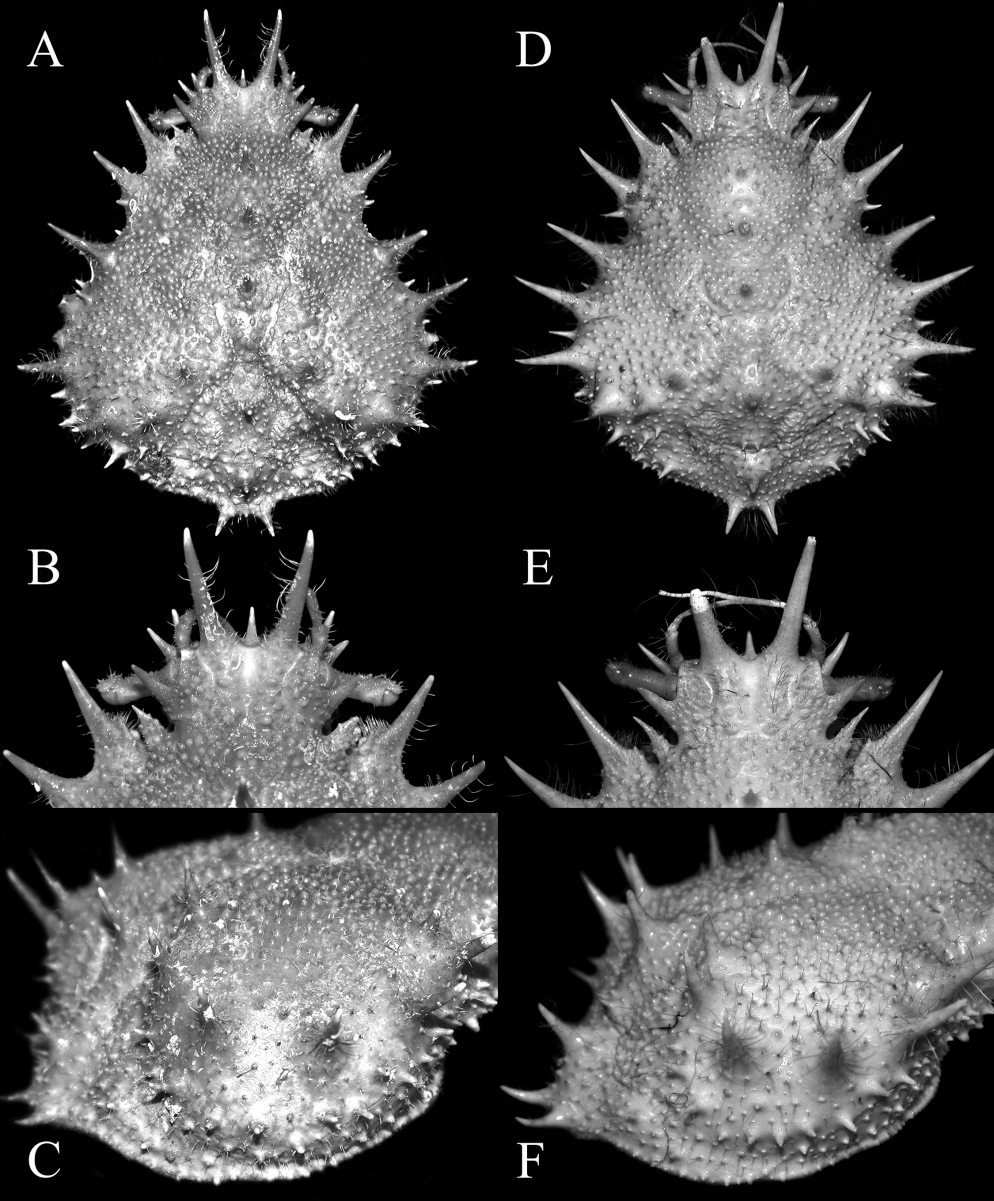
**A–C** *Paramaya mulli* sp. n., holotype male (70.4 × 61.4 mm) (CASAU), India **D–F** *P. spinigera* (De Haan, 1837), male (73.6 × 55.3 mm) (ZRC 1999.738), Taiwan **A, D** dorsal view of carapace **B, E** frontal part of carapace **C, F** lateral view of branchial region of carapace.

**Figure 4.**
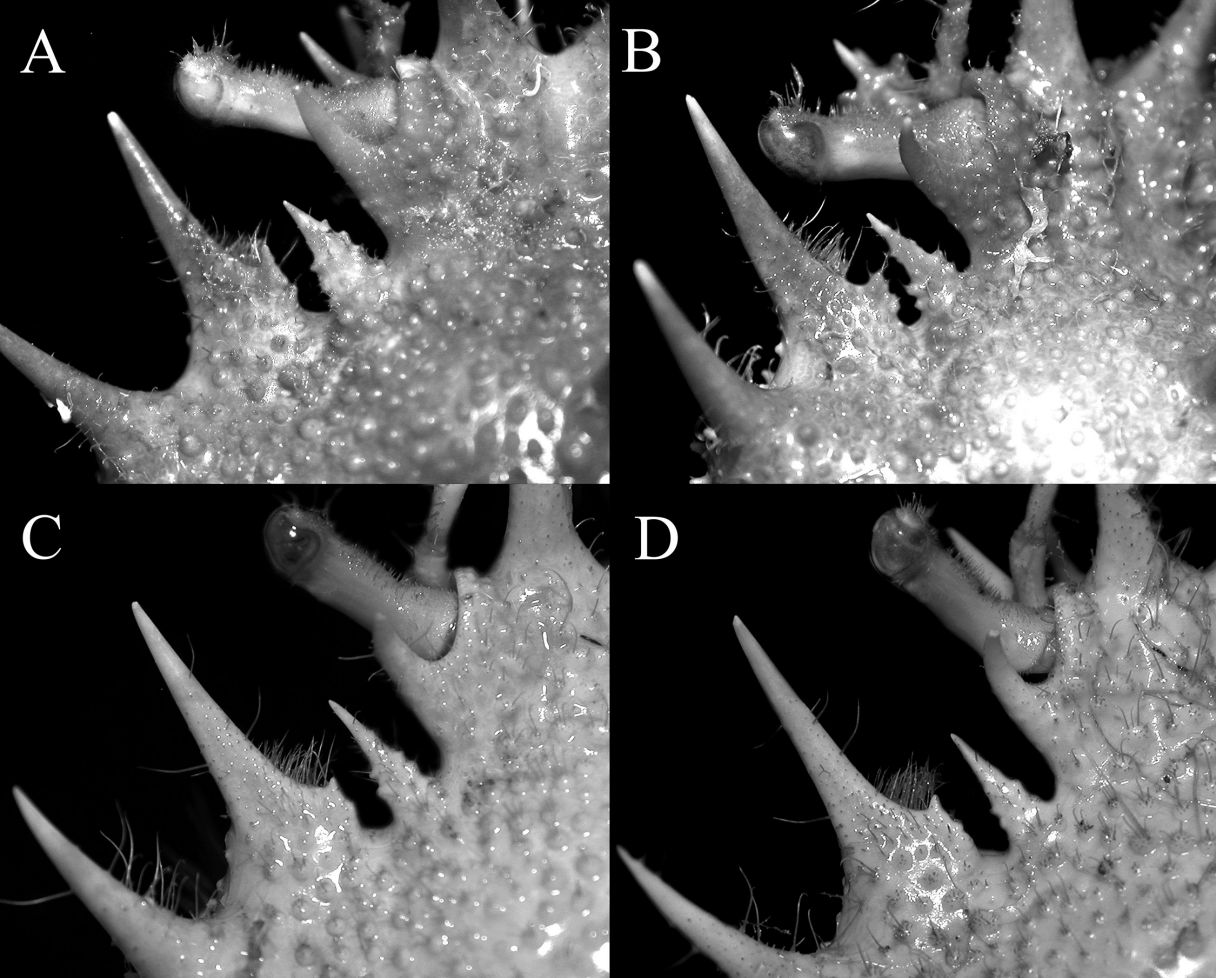
Intercalated spine **A** *Paramaya mulli* sp. n., holotype male (70.4 × 61.4 mm) (CASAU), India **B** *P. mulli* sp. n., paratype female (40.0 × 33.5 mm) (CASAU), India **C** *P. spinigera* (De Haan, 1837), male (73.6 × 55.3 mm) (ZRC 1999.738), Taiwan **D** *P. spinigera* (De Haan, 1837), male (85.0 × 66.4 mm) (ZRC 1999.738), Taiwan.

**Figure 5.**
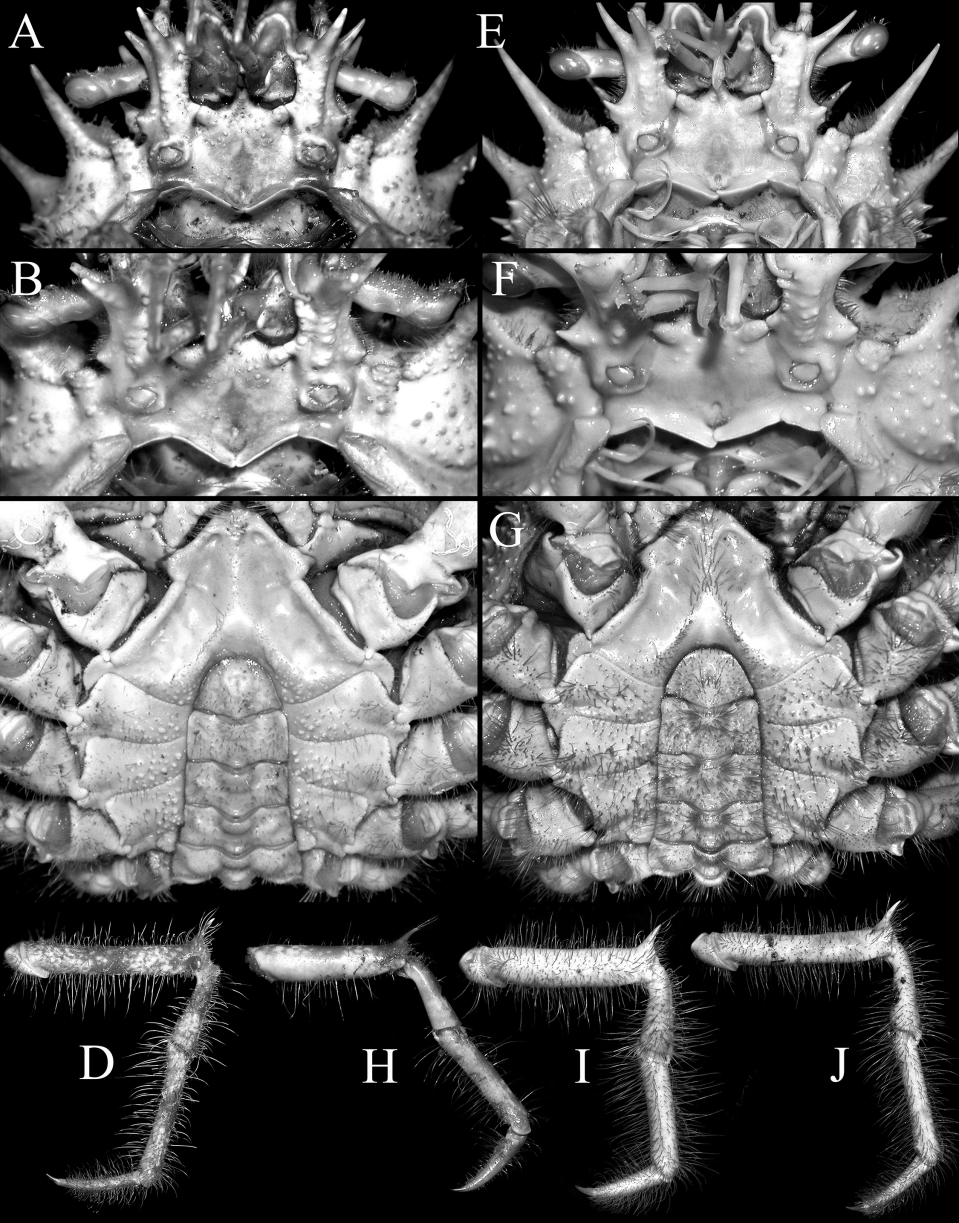
**A–D** *Paramaya mulli* sp. n., holotype male (70.4 × 61.4 mm) (CASAU), India **H** *P. mulli* sp. n., paratype female (40.0 × 33.5 mm) (CASAU), India **E–G, I** *P. spinigera* (De Haan, 1837), male (73.6 × 55.3 mm) (ZRC 1999.738), Taiwan **J** *P. spinigera* (De Haan, 1837), male (85.0 × 66.4 mm) (ZRC 1999.738), Taiwan **A, B, E, F** epistome, basal antennal article and antennules **C, G** thoracic sternum and pleon **D–J** right fourth ambulatory leg.

**Figure 6.**
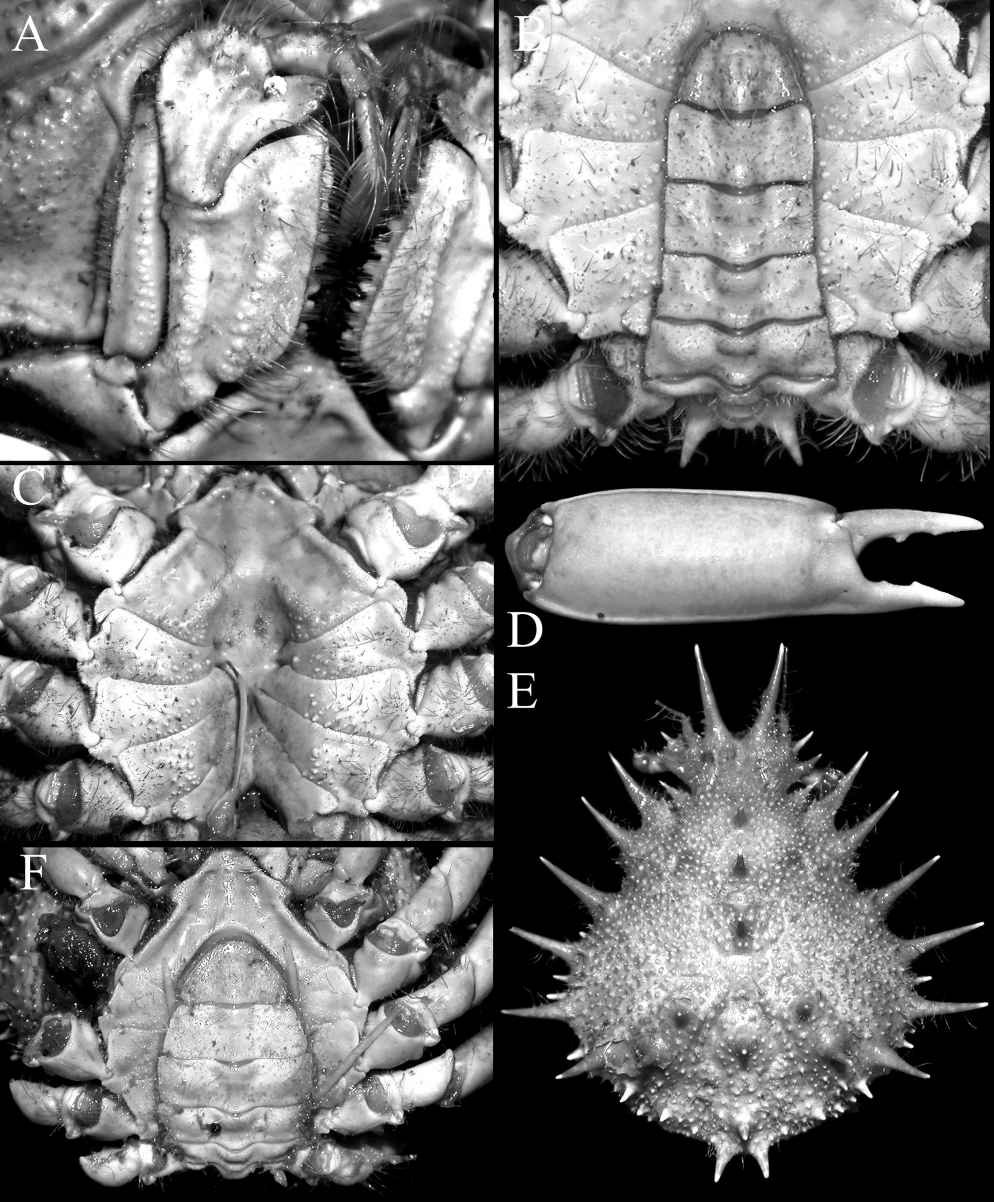
**A–D** *Paramaya mulli* sp. n., holotype male (70.4 × 61.4 mm) (CASAU), India **E, F** *P. mulli* sp. n., paratype female (40.0 × 33.5 mm) (CASAU), India **A** right third maxilliped **B** thoracic sternum and pleon; C, sternopleonal cavity **D** outer view of right chela **E** dorsal view of carapace **F** thoracic sternum and pleon.

**Figure 7.**
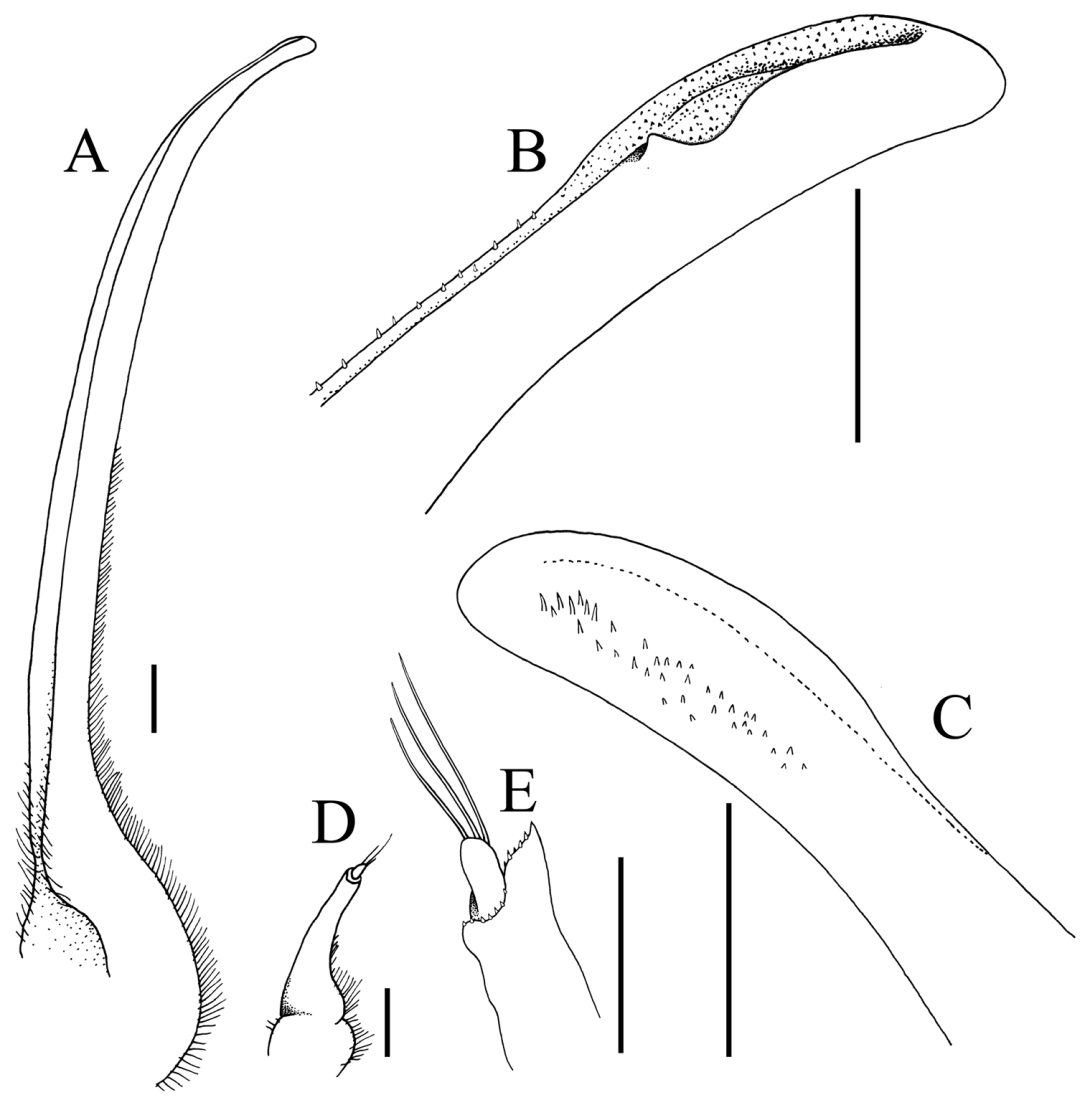
*Paramaya mulli* sp. n., holotype male (70.4 × 61.4 mm) (CASAU), India **A** left G1 (ventral view) **B** distal part of left G1 (ventral view) **C** distal part of left G1 (dorsal view) **D** left G2 **E** distal part of left G2. Scale bars: **A, D** 1.0 mm **B, C, E** 0.5 mm.

Figs 1 , 2A–C , 3A–C , 4A, B , 5A–D, H , 6 , 7

Maia spinigera – Alcock 1895: 239; Alcock and Anderson 1898: pl. 34, fig. 3.
Maja spinigera” – Ng and Richer de Forges 2015: 156, fig. 22B–D.
Non Pisa (Paramaya) spinigera De Haan, 1837.

##### Material examined.

Holotype: male (70.4 × 61.4 mm) (CASAU), Pazhayar fish landing centre, facing Bay of Bengal, Tamil Nadu, India, 11°21'11.5"N, 79°45'26.3"E, from trawls, coll. M. Prema and S. Ravichandran, 7 February 2018. Paratype: 1 female (40.0 × 33.5 mm) (CASAU), same data as holotype.

##### Comparative material examined.

*Paramaya spinigera* (De Haan, 1837): 7 males (85.0 × 66.4 mm, 78.2 × 62.1 mm, 73.6 × 55.3 mm, 68.3 × 53.4 mm, 73.8 × 58.4 mm, 62.8 × 49.0 mm, 72.8 × 57.3 mm), 1 ovigerous female (63.0 × 48.6 mm) (ZRC 1999.738), Longtong, near Keelung, northern Taiwan, in tangle nets for lobsters, coll. S-H Wu, May 1999. For other material of *Paramaya* species, see Ng and Richer de Forges (2015).

##### Diagnosis.

Pseudorostral horns relatively short (Figs 2A, 3A, B); hepatic, lateral and branchial spines long; median row with 5 spines: 3 gastric, 1 cardiac, 1 intestinal; 2 spines on posterior carapace margin (Figs 2A, 3A); adult branchial region distinctly swollen (Fig. 3A, C); intercalated tooth on carapace relatively broad (Figs 3B, 4A, B); epistome quadrate (Fig. 5A, B); surface of thoracic sternum not prominently setose, with numerous prominent rounded granules (Figs 5C, 6B); chela of adult male with distinct carina on dorsal and ventral margins (Figs 2A, 6D); ambulatory meri in adult males relatively slender, long (Figs 2A, 5D); G1 gently curved, dorsal projection on the sub distal part low, tip distinctly rounded (Fig. 7A–C).

**Colour**. Freshly obtained specimens have the dorsal surfaces orangish-red, with red and white bands on ambulatory legs; chelipeds yellowish-orange with white fingers; ventral surfaces white with patches of orange (Fig. 1).

##### Etymology.

The species is named after the famous Mulli plant in Tamil mythology, from the classic poetic work Kurunthogai. Mulli is a coastal plant (*Spinifex littoreus* (Burm.f.) Merr., family Poaceae) with very sharp spines (mull is the Tamil word for spiny), a character shared with the present species. The name is used as a noun in apposition.

##### Remarks.

Compared to *P. spinigera*, the branchial region of adult male *P. mulli* sp. n. is more swollen (Fig. 3A, C) (versus gently convex in *P. spinigera*; Fig. 3D, F); the intercalated tooth on the carapace is relatively broader (Figs 3B, 4A, B) (versus more acutely triangular in *P. spinigera*; Figs 3E, 4C, D); the epistome is more quadrate (Fig. 5A, B) (versus more transversely rectangular in *P. spinigera*; Fig. 5E, F); the surface of the male thoracic sternum, especially the areas adjacent to the sternopleonal cavity is distinctly granulated with scattered setae (Fig. 5C) (versus surfaces weakly granulate with dense setae in *P. spinigera*; Fig. 5G); and the G1 has the dorsal projection on the subdistal part relatively small with the tip more prominently rounded (Fig. 7A–C) (versus dorsal projection on the subdistal part more developed with the tip gently tapering in *P. spinigera*; Ng and Richer de Forges 2015: fig. 23A–C).

There is variation in the proportions of the ambulatory legs of *Paramaya* species. In the series of specimens of *P. spinigera* on hand, females generally have relatively shorter ambulatory legs compared to males. In addition, for each sex, smaller specimens have proportionately shorter and stouter legs (Fig. 5I) compared to larger ones (Fig. 5J). When comparing the holotype male *P. mulli* sp. n. (70.4 × 61.4 mm, CASAU) with a similar size male of *P. spinigera* from Taiwan (73.6 × 55.3 mm, ZRC 1999.738), the merus, propodus and dactylus of *P. mulli* sp. n. (Fig. 5D) is significantly more slender and longer than that of *P. spinigera* (Fig. 5I). In larger male specimens of *P. spinigera* from Taiwan (85.0 × 66.4 mm, ZRC 1999.738), the merus is proportionately longer but is still relatively stouter (Fig. 5J). Females of both species have relatively shorter and stouter ambulatory legs compared to males (Fig. 5H).

Ng and Richer de Forges (2015: 156) noted that the specimen mentioned and figured by Alcock (1895) and Alcock and Anderson (1898) as “*P. spinigera*” has short ambulatory meri, but this is probably because this specimen was small; and the larger specimen from Sri Lanka they examined a photograph has proportionately longer ambulatory legs. As discussed above, the proportions of the ambulatory meri is clearly correlated with size. Noteworthy is that the Sri Lankan specimen also has relatively more inflated branchial regions, and as such, is almost certainly conspecific with what is described here as *P. mulli* sp. n.

The distinctly granulated thoracic sternum of *P. mulli* sp. n. (Fig. 5C) allies the species with *P. ouch* (Ng and Richer de Forges 2015: fig. 50B), but in *P. ouch*, the branchial region is not distinctly swollen, and the pseudorostral and carapace spines are proportionately longer across all size ranges in both sexes (cf. Ng and Richer de Forges 2015: figs 21E, F, 37B) (versus branchial regions more swollen and the spines are proportionately shorter in *P. mulli* sp. n.; Figs 2A, 3A–C, 6E). In addition, the distal part of the G1 in *P. ouch* is more strongly curved (Ng and Richer de Forges 2015: fig. 23D) with the dorsal projection on the subdistal part prominent and the tip is relatively more angular (Ng and Richer de Forges 2015: fig. 23E, F) (versus distal part of G1 less curved with the dorsal projection low and tip rounded in *P. mulli* sp. n.; Fig. 7A–C). Compared to *P. mulli* sp. n., *P. coccinea* has proportionately longer pseudorostral and carapace spines with the branchial region not distinctly swollen (Ng and Richer de Forges 2015: figs 22A, 37C), the male thoracic sternum is almost smooth with the granules low (Ng and Richer de Forges 2015: fig. 50C) and the dorsal projection on the subdistal part of the G1 is prominent with the tip relatively more angular (Ng and Richer de Forges 2015: fig. 23H, I) (cf. pseudorostral and carapace spines proportionately shorter, the male thoracic sternum is distinctly granulated and the dorsal projection on the G1 subdistal part is low with the tip rounded; Figs 2A, 3A–C, 5C, 6E, 7A–C). In addition, even though the holotype male and only known specimen of *P. coccinea* is about the same size as the holotype male of *P. mulli* sp. n., the ambulatory meri are proportionately much longer (cf. Ng and Richer de Forges 2015: figs 22A, 70B) (versus distinctly shorter in *P. mulli* sp. n.; Figs 2A, 5D).

Like other *Paramaya* species, the preferred habitat of *P. mulli* sp. n. is probably relatively steep and rocky areas that are difficult to sample except with tangle nets (see Ng et al. 2009, Mendoza et al. 2010). As such, normal fishery operations using trawls are less likely to obtain them and could explain their apparent rarity in Indian waters.

## Supplementary Material

XML Treatment for Paramaya

XML Treatment for Paramaya mulli
